# Supplementary material for: Quantitative Description of the Surface Tension Minimum in a Two-Component Surfactant System
Source: Langmuir. 2025 Oct 15;41(42):28521–35. doi: 10.1021/acs.langmuir.5c03750 (PMC12573795; doi:10.1021/acs.langmuir.5c03750)
Supplement: Supplementary file 1 [file la5c03750_si_001.pdf]

Supporting Information for: Quantitative description of the surface tension minimum in a two-component surfactant system

Edgar M. Blokhuis*

Colloid and Interface Science, Leiden Institute of Chemistry,
Leiden University, 2300 RA Leiden, The Netherlands

E-mail: e.blokhuis@chem.leidenuniv.nl

Table of contents:

A Statistical Thermodynamic derivation of the Langmuir model:

1 single surfactant type

2 surfactant mixture (equal surface size)

3 surfactant mixture (unequal surface size)

4 ionic surfactant

B Outline computer program non-ionic surfactant mixture

C Micellar composition: composition range example

1 Statistical Thermodynamic derivation of the Langmuir model – single surfactant type

We discuss the Statistical Thermodynamic derivation of the Langmuir model for the adsorption of a single surfactant type and extend it to mixtures. First, we consider adsorption of a single surfactant type. Surfactant interactions on the surface are regarded as purely repulsive and taken into account by limiting the total amount of available surface positions to a maximum number $N_{\rm max}$. When a surfactant molecule adsorbs at the surface from a (reference) bulk solution, a certain adsorption energy, which we shall denote as $\Delta E_{\rm s}$ is associated with it. The grand canonical partition function Ξ can then be explicitly evaluated to give:

$$\Xi = \sum_{N=0}^{N_{\rm max}} W(N)\, e^{-(\Delta E_{\rm s}+\mu_{\rm s}^{\circ}-\mu_{\rm s})N/k_{\rm B}T} = \sum_{N=0}^{N_{\rm max}} \binom{N}{N_{\rm max}} x^N = (1+x)^{N_{\rm max}}\,, \tag{1}$$

where $W(N)$ is the number of ways N molecules can be distributed over $N_{\rm max}$ available positions and where x is defined as

$$x \equiv \exp\left[\,(\mu_{\rm s}-\mu_{\rm s}^{\circ}-\Delta E_{\rm s})/k_{\rm B}T\,\right]. \tag{2}$$

This gives for the surface grand free energy

$$\Omega = -k_{\rm B}T\,\ln(\Xi) = -N_{\rm max}\,k_{\rm B}T\,\ln(1+x)\,, \tag{3}$$

The average number of surfactants $N_{\rm s}$ adsorbed at the surface is obtained from the grand free energy by differentiation:

$$N_{\rm s} = -\left(\frac{\partial\Omega}{\partial\mu_{\rm s}}\right)_T = N_{\rm max}\,\frac{x}{1+x}\,. \tag{4}$$

In a more continuous form, in which the (discrete) number of surfactants adsorbed $N_{\rm s}$ is replaced by the surfactant adsorption Γ, this result leads to the well-known *Langmuir isotherm*

$$\frac{\Gamma}{\Gamma_\infty} = \frac{x}{1+x}\,. \tag{5}$$

The surface grand free energy is essentially the contribution to the total surface tension σ due to the presence of surfactants. In a continuous form this gives:

$$\sigma = \sigma_0 - k_{\rm B}T\,\Gamma_\infty\,\ln(1+x)\,. \tag{6}$$

This expression is a direct result of the Statistical Thermodynamic evaluation of the partition function but can also be derived by integration of the *Gibbs adsorption equation*

$$\left(\frac{\partial\sigma}{\partial\mu_{\rm s}}\right)_T = -\Gamma\,. \tag{7}$$

using the Langmuir isotherm in Eq.(5):

$$\sigma = -\int d\mu_{\rm s}\,\Gamma = -k_{\rm B}T\,\Gamma_\infty \int dx\,\frac{1}{1+x} = \sigma_0 - k_{\rm B}T\,\Gamma_\infty\,\ln(1+x)\,. \tag{8}$$

2 Statistical Thermodynamic derivation of the Langmuir model – surfactant mixture (equal surface size)

Next, we consider adsorption of a surfactant mixture. Initially, we shall assume that all $N_{\rm max}$ available positions are equally accessible to both species, with the only distinction being that species a gains an energy $\Delta E_{\rm s,a}$ upon adsorption and species b an energy $\Delta E_{\rm s,b}$. In that situation, the grand canonical partition function is evaluated as:

$$\begin{aligned}
\Xi &= \sum_{N_a=0}^{N_{\rm max}} \sum_{N_b=0}^{N_{\rm max}-N_a} W(N_a, N_b)\, e^{-(\Delta E_{\rm s,a}+\mu_{\rm a}^{\circ}-\mu_a)N_a/k_{\rm B}T}\, e^{-(\Delta E_{\rm s,b}+\mu_{\rm b}^{\circ}-\mu_b)N_b/k_{\rm B}T} \\
&= \sum_{N_a=0}^{N_{\rm max}} \binom{N_a}{N_{\rm max}} x_a^{N_a} \sum_{N_b=0}^{N_{\rm max}-N_a} \binom{N_b}{N_{\rm max}-N_a} x_b^{N_b} \\
&= \sum_{N_a=0}^{N_{\rm max}} \binom{N_a}{N_{\rm max}} x_a^{N_a}\, (1+x_b)^{N_{\rm max}-N_a} \\
&= (1+x_b)^{N_{\rm max}}\, (1+\frac{x_a}{1+x_b})^{N_{\rm max}} = (1+x_a+x_b)^{N_{\rm max}}\,, \qquad (9)
\end{aligned}$$

where $W(N_a, N_b)$ is the number of ways N_a molecules of type a and N_b molecules of type b can be distributed over $N_{\rm max}$ available positions. The parameters x_a and x_b are defined as

$$x_a \equiv \exp\left[\,(\mu_a - \mu_{\rm a}^{\circ} - \Delta E_{\rm s,a})/k_{\rm B}T\,\right] \qquad \text{and} \qquad x_b \equiv \exp\left[\,(\mu_b - \mu_{\rm b}^{\circ} - \Delta E_{\rm s,b})/k_{\rm B}T\,\right]. \quad (10)$$

This gives for the surface grand free energy

$$\Omega = -k_{\rm B}T\, \ln(\Xi) = -N_{\rm max}\; k_{\rm B}T\, \ln(1+x_a+x_b)\,. \quad (11)$$

The average number of surfactants N_a and N_b adsorbed at the surface are obtained from the grand free energy by differentiation:

$$N_a = -\left(\frac{\partial\Omega}{\partial\mu_a}\right)_T = \frac{N_{\rm max}\; x_a}{1+x_a+x_b} \qquad \text{and} \qquad N_b = -\left(\frac{\partial\Omega}{\partial\mu_b}\right)_T = \frac{N_{\rm max}\; x_b}{1+x_a+x_b}\,. \quad (12)$$

In a more continuous form, in which the (discrete) number of surfactants adsorbed is replaced by the surfactant adsorption Γ, this result leads to the *Langmuir isotherm* for mixtures:

$$\frac{\Gamma_a}{\Gamma_\infty} = \frac{x_a}{1+x_a+x_b} \qquad \text{and} \qquad \frac{\Gamma_b}{\Gamma_\infty} = \frac{x_b}{1+x_a+x_b}\,. \quad (13)$$

The surface tension is read off from the surface grand free energy:

$$\sigma = \sigma_0 - k_{\rm B}T\; \Gamma_\infty\, \ln(1+x_a+x_b)\,. \quad (14)$$

3 Statistical Thermodynamic derivation of the Langmuir model – surfactant mixture (unequal surface size)

Finally, we again consider adsorption of a surfactant mixture but allow for the fact that one species (species a) takes up more of the available area than the other (species b) by a factor of $\beta \geqslant 1$. In that case, the grand canonical partition function is evaluated as:

$$\begin{aligned}
\Xi &= \sum_{N_a=0}^{N_{\max}} \sum_{N_b=0}^{\beta(N_{\max}-N_a)} W(N_a, N_b)\, e^{-(\Delta E_{\mathrm{s,a}}+\mu_{\mathrm{a}}^{\circ}-\mu_a)N_a/k_{\mathrm{B}}T}\, e^{-(\Delta E_{\mathrm{s,b}}+\mu_{\mathrm{b}}^{\circ}-\mu_b)N_b/k_{\mathrm{B}}T} \\
&= \sum_{N_a=0}^{N_{\max}} \binom{N_a}{N_{\max}} x_a^{N_a} \sum_{N_b=0}^{\beta(N_{\max}-N_a)} \binom{N_b}{\beta(N_{\max}-N_a)} x_b^{N_b} \\
&= \sum_{N_a=0}^{N_{\max}} \binom{N_a}{N_{\max}} x_a^{N_a}\, (1+x_b)^{\beta(N_{\max}-N_a)} \\
&= (1+x_b)^{\beta N_{\max}}\, (1+\frac{x_a}{(1+x_b)^{\beta}})^{N_{\max}} = (x_a+(1+x_b)^{\beta})^{N_{\max}}\,. \qquad (15)
\end{aligned}$$

This gives for the surface grand free energy

$$\Omega = -k_{\mathrm{B}}T\, \ln(\Xi) = -N_{\max}\, k_{\mathrm{B}}T\, \ln(x_a+(1+x_b)^{\beta})\,. \qquad (16)$$

The average number of surfactants adsorbed at the surface is again obtained from the grand free energy by differentiation [1, 2]:

$$\frac{N_a}{N_{\max}} = \frac{\Gamma_a}{\Gamma_{a,\infty}} = \frac{x_a}{x_a+(1+x_b)^{\beta}} \qquad \text{and} \qquad \frac{N_b}{\beta N_{\max}} = \frac{\Gamma_b}{\Gamma_{b,\infty}} = \frac{x_b\,(1+x_b)^{\beta-1}}{x_a+(1+x_b)^{\beta}}\,. \qquad (17)$$

The surface tension is again read off from the surface grand free energy:

$$\sigma = \sigma_0 - k_{\mathrm{B}}T\, \Gamma_{a,\infty}\, \ln(x_a+(1+x_b)^{\beta})\,, \qquad (18)$$

where $\beta = \Gamma_{b,\infty}/\Gamma_{a,\infty}$.

4 Statistical Thermodynamic derivation of the Langmuir model – ionic surfactant

In the treatment of ionic surfactants, we have assumed that the surfactants are fully dissociated in the bulk solution but fully associated at the surface. We discuss the consequences of this assumption in the context of the Langmuir model. We only consider a single non-ionic surfactant type in the presence of added salt. The extension to surfactant mixtures follows in analogy to the treatment of non-ionic surfactant mixtures with the replacement discussed below.

For notational convenience, we assume that the solution consists of SDS surfactant molecules with NaCl as added salt. Three type of ions (DS^-, Na^+, Cl^-) are then in solution. At the surface, only the associated SDS is present. Again, surfactant interactions on the surface are regarded as purely repulsive and taken into account by limiting the total amount of available surface positions to a maximum number $N_{\rm max}$. The adsorption energy for a DS^- ion or for a Na^+ ion to adsorb to the surface is denoted as $\Delta E_{\rm s,DS}$ or $\Delta E_{\rm s,Na}$, respectively.

The grand canonical partition function Ξ can then be explicitly evaluated to give:

$$\begin{aligned} \Xi &= \sum_{N=0}^{N_{\rm max}} W(N)\, e^{-(\Delta E_{\rm DS}+\mu^\circ_{\rm DS}-\mu_{\rm DS}+\Delta E_{\rm Na}+\mu^\circ_{\rm Na}-\mu_{\rm Na})N/k_{\rm B}T} \qquad (19) \\ &= \sum_{N=0}^{N_{\rm max}} \binom{N}{N_{\rm max}} (x_{\rm DS}\, x_{\rm Na})^N = (1+x_{\rm DS}\, x_{\rm Na})^{N_{\rm max}} \end{aligned}$$

where x_i ($i=$ DS^-, Na^+) is defined as

$$x_i \equiv \exp\left[\,(\mu_{\rm i}-\mu^\circ_{\rm i}-\Delta E_{\rm s,i})/k_{\rm B}T\,\right]. \qquad (20)$$

This gives for the surface grand free energy

$$\Omega = -k_{\rm B}T\,\ln(\Xi) = -N_{\rm max}\; k_{\rm B}T\,\ln(1+x_{\rm DS}\, x_{\rm Na})\,, \qquad (21)$$

The average number of surfactants adsorbed at the surface is obtained from the grand free energy by differentiation with respect to the respective chemical potential. In a continuous form, this leads to the following adaptation of the Langmuir isotherm

$$\Gamma_{\rm DS^-} = \Gamma_{\rm Na^+} = \Gamma_\infty \frac{x_{\rm DS}\, x_{\rm Na}}{1+x_{\rm DS}\, x_{\rm Na}}\,. \qquad (22)$$

The surface grand free energy is essentially the contribution to the total surface tension σ due to the presence of surfactants. In a continuous form it is given by

$$\sigma = \sigma_0 - k_{\rm B}T\;\Gamma_\infty\,\ln(1+x_{\rm DS}\, x_{\rm Na})\,. \qquad (23)$$

When we compare these expressions to the analogous expressions for a non-ionic surfactant, we see that x is replaced by the product $x_{\rm DS}\, x_{\rm Na}$. It is easy to verify that the analogous expressions for a *mixture* of SDS and contaminant are derived from the non-ionic surfactant formulas using the same replacement.

5 Outline computer program non-ionic surfactant mixture

Units: concentration in mol/L, surface tension in mN/m, adsorption in mol/m^2, K in L/mol

Constants: $\sigma_0 = 72.0$, $RT = 8.314 \cdot 298 \cdot 10^3$, $v_0 = 1/55.3$

Parameters: $\Gamma_{a,\infty}$, K_a, x_0^a, $\Gamma_{b,\infty}$, K_b, x_0^b, m, α

Define $\beta = \Gamma_{b,\infty}/\Gamma_{a,\infty}$

i) Micellar composition: two types of micelle: $p_{\min} = m_a$ and $p_{\max} = m_a$

ii) Micellar composition: composition range: $p_{\min} = m_a$ and $p_{\max} = m - 1$

iii) Micellar composition: mixing entropy: $p_{\min} = 1$ and $p_{\max} = m - 1$

Loop 1: vary $x_{1,a}$ between 0 and $x_{0,a}$

Define $x_{1,b,\min} = 0$

Define $x_{1,b,\max} = \alpha\, x_{1,a}$ (we assume $\alpha < 1$, if $\alpha > 1$ then reverse roles of a and b)

Loop 2: iterate to find $x_{1,b}$ until desired accuracy for Δ

Define $x_{1,b} = \frac{1}{2}(x_{1,b,\min} + x_{1,b,\max})$

Define $x_{m,a,\text{tot}} = m\,(x_{1,a}/x_{0,a})^m$

Define $x_{m,b,\text{tot}} = 0$ (regular surfactants with mixing entropy: $x_{m,b,\text{tot}} = m\,(x_{1,b}/x_{0,b})^m$)

Loop 3: vary p_a by 1 from $p_{\min}$ to $p_{\max}$

Define $p_b = \beta\,(m - p_a)$ (regular surfactants with mixing entropy: $p_b = m - p_a$)

Define $W_{\text{ent}} = m!\,/\,(\,m\ p_a!\ (m - p_a)!\,)$ (no mixing entropy: $W_{\text{ent}} = 1$)

Define $x_{m,a,\text{tot}} = x_{m,a,\text{tot}} + p_a\,(x_{1,a}/x_{0,a})^{p_a}\,(x_{1,b}/x_{0,b})^{p_b}\ W_{\text{ent}}$

Define $x_{m,b,\text{tot}} = x_{m,b,\text{tot}} + p_b\,(x_{1,a}/x_{0,a})^{p_a}\,(x_{1,b}/x_{0,b})^{p_b}\ W_{\text{ent}}$

End Loop 3

Define $x_{\text{tot,a,estimate}} = x_{1,a} + x_{m,a,\text{tot}}$

Define $x_{\text{tot,b,estimate}} = x_{1,b} + x_{m,b,\text{tot}}$

Define $\Delta = x_{\text{tot,b,estimate}} - \alpha\, x_{\text{tot,a,estimate}}$

if $\Delta > 0$ then $x_{1,b,\max} = x_{1,b}$ else $x_{1,b,\min} = x_{1,b}$

End Loop 2

Define $c_s = (x_{\text{tot,a,estimate}} + x_{\text{tot,b,estimate}})/v_0$

Define $c_{1,a} = x_{1,a}/v_0$

Define $c_{1,b} = x_{1,b}/v_0$

Define $\sigma = \sigma_0 - RT\ \Gamma_{a,\infty}\,\ln(K_a\,c_{1,a} + (1 + K_b\,c_{1,b})^{\beta})$ $\quad(\beta \geqslant 1)$

Define $\sigma = \sigma_0 - RT\ \Gamma_{b,\infty}\,\ln(K_b\,c_{1,b} + (1 + K_a\,c_{1,a})^{1/\beta})$ $\quad(\beta \leqslant 1)$

Write $(c_s,\ \sigma)$

End Loop 1

6 Micellar composition: composition range example

In the Figure below, the distribution corresponding to the solid black curve in Figure 2b in the main text ($m_a = 35$ .. 50) is shown. The distribution shows the unnatural cut-off at the minimum micellar size ($m_a = 35$) that is avoided in the entropy approach.

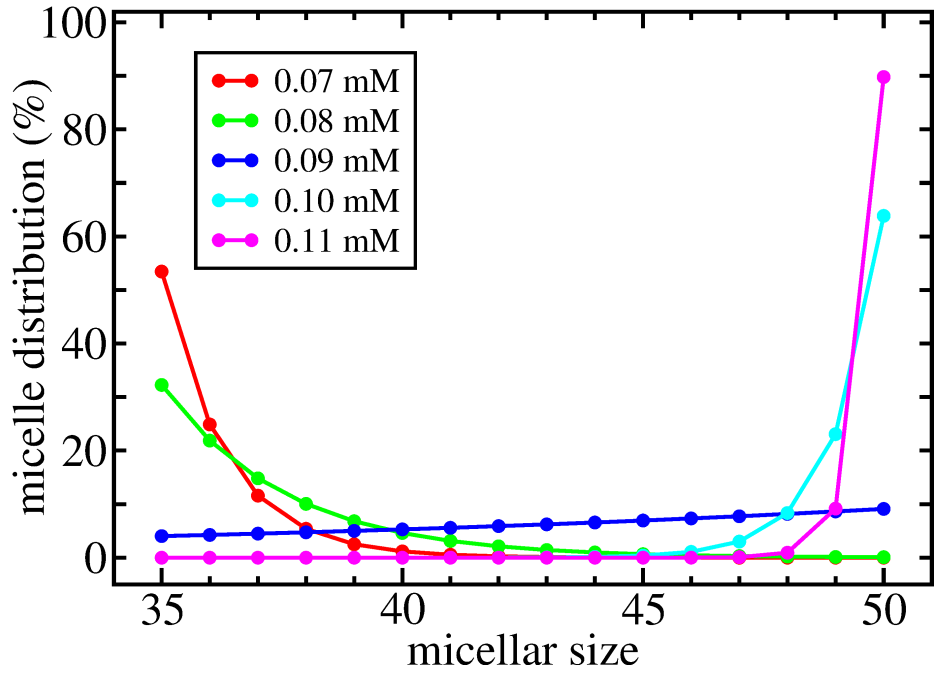

Figure 1: Micellar distributions for the $C_{12}E_8$ + contaminant system as a function of micellar size (m_a) for a number of surfactant concentrations. This example corresponds to the solid black curve in Figure 2b in the main text ($m_a = 35$ .. 50).

References

[1] Van Assche, T.R.C.; Baron, G.V.; Denayer, J.F.M. An explicit multicomponent adsorption isotherm model: accounting for the size-effect for components with Langmuir adsorption behavior *Adsorption* **2018**, 24, 517-530.

[2] Van Assche, T.R.C.; Baron, G.V.; Denayer, J.F.M. Properties of an explicit, thermodynamic consistent model as intermediate between extended Langmuir and IAST mixture adsorption models *Adsorption* **2024**, 30, 351-361.
